# Surgical impact of laparoscopic and laparotomic elective ovariectomy on inflammatory and oxidative stress biomarkers in anestrus dogs

**DOI:** 10.1111/vsu.70070

**Published:** 2026-01-03

**Authors:** Alfonso Calabria, Chiara Del Prete, Maria Pia Pasolini, Veronica Palumbo, Maria Anna Nappo, Silvia Ammirati, Davide Ciccarelli, Fabiana Micieli, Michal Andrzej Kosior, Carmine Carbone, Natascia Cocchia

**Affiliations:** ^1^ Department of Veterinary Medicine and Animal Production Federico II University of Naples Naples Italy; ^2^ ASL Napoli 3 sud Naples Italy; ^3^ Freelancer Naples Italy

## Abstract

**Objective:**

To compare the systemic inflammatory and oxidative stress responses after elective ovariectomy via open surgery (OPEN) or laparoscopy (LAP) in anestrus bitches.

**Study design:**

Prospective, blinded, randomized clinical trial.

**Animals**

A total of 26 healthy bitches.

**Methods:**

A total of 26 healthy bitches in anestrus were randomly assigned to undergo either LAP or OPEN ovariectomy (*n* = 13 per group). Blood samples were collected at four timepoints (preoperative‐Tpre, 2 h‐T2, 24 h‐T24, and 7 days postoperative‐T7) for quantification of interleukin‐6 (IL‐6) and paraoxonase‐1 (PON‐1), biological antioxidant potential (BAP), and reactive oxygen metabolites (d‐ROMs).

**Results:**

IL‐6 levels were higher in the OPEN group at Tpre, T24 and T7, while the LAP group exhibited a transient peak at T2 with a return to baseline by T7. PON‐1 levels decreased in both groups at T2 but remained lower in the LAP group at T24 and T7. d‐ROM levels were higher in the OPEN group, with an increase through T7, whereas the LAP group showed only a transient peak at T24. BAP levels increased in the OPEN group, but not in the LAP group by T7.

**Conclusion:**

LAP ovariectomy induced a milder and more transient inflammatory and oxidative response compared to OPEN surgery in bitches, likely due to reduced surgical trauma.

**Clinical significance:**

This study included only animals in anestrus. This is the first application of both canine‐specific biomarkers of inflammation and canine‐validated biomarkers of oxidative stress to compare techniques of elective canine spaying. These findings suggest that minimally invasive ovariectomy attenuates inflammatory and oxidative stress responses compared to OPEN technique in dogs.

## 1 INTRODUCTION

Surgical sterilization of dogs and cats remains the most common surgical procedure.^1^ Research into the most valuable surgical procedure continues to arouse the interest of the scientific community. Surgical sterilization of female dogs via laparoscopic techniques has gained popularity for its numerous benefits: shorter hospitalization, lower incidence of postoperative complications, and a reduction in inflammation and surgical trauma.^1^, ^2^, ^3^, ^4^ Every surgical trauma results in a broad spectrum of adverse alterations to normal body homeostasis that involve the activation of inflammatory, endocrine, metabolic, and immunological mediators^5^ that are collectively referred to as surgical stress and have been recognized as a trigger of inflammatory and oxidative stress (OS) responses.^6^ The inflammatory response is a key trauma‐response defense mechanism that initiates tissue repair, but if it is prolonged or uncontrolled, it can cause adverse effects such as pain or immunosuppression.^7^, ^8^ The inflammatory response related to surgical or anesthetic trauma can be defined as surgical inflammation, which involves a complex set of pathophysiological mechanisms^9^, ^10^ and can activate cytokine inflammatory cascades.^9^, ^11^

Numerous biomarkers have been tested to quantify the inflammatory response to surgical trauma.^11^ Interleukin 6 (IL‐6) is recognized as a key proinflammatory cytokine, and its serum concentration is directly related to the activation of the inflammatory response.^12^ Paraoxonase‐1 (PON‐1), on the other hand, is a serum enzyme with antioxidant activity that could be used as an inflammation marker and a prognostic indicator. A reduction in PON‐1 activity has been reported to be inversely related to the severity of inflammation and clinical outcomes. Although this remains controversial it has also been reported in dogs.^13^

While laparotomy has previously been identified as a source of OS,^14^ the duration and severity of OS that is induced by creation of pneumoperitoneum in laparoscopy is unknown.^15^, ^16^, ^17^ The OS response, characterized by perturbation of the balance between pro‐oxidants and antioxidants due to an imbalance between free radical production and antioxidant defenses,^18^ is an integral part of the surgical stress response.^3^ OS during surgery results from two main mechanisms.^7^ First, tissue damage and the resulting hypoxia activate polymorph nuclear leukocytes and monocytes, leading to increased production of reactive oxygen species (ROS). Second, in the postoperative period, there is a redistribution and an increase in the consumption of antioxidants.^18^ Sources of OS during surgery include laparotomy, severe ischemic conditions, and the activation of inflammatory cells.^7^, ^19^ Excess ROS can cause oxidative damage to proteins, lipids and DNA, resulting in cell toxicity.^20^, ^21^ Scientific research, therefore, aims to measure the levels of ROS and antioxidant potential during the perioperative period^22^ to define true markers of OS and to develop strategies for reducing oxidative damage.^20^, ^23^ As biomarkers of OS biological antioxidant potential (BAP) and derivative compounds of reactive oxygen metabolites (d‐ROMs) have been validated in the dog.^23^ The d‐ROMs test have been recognized as useful biomarkers in several clinical conditions, including tumors and environmental stress in dogs.^24^ It measures the overall oxidant capacity of plasma against N,N‐diethylparaphenylendiamine in an acidic buffer. Hydroperoxides are primarily responsible for this oxidant capacity, with minor contributions from other oxidant factors.^23^ The BAP test evaluates the plasma antioxidant biological potential as the capacity of the plasma sample to reduce ferric ions to ferrous ions. The major component of plasma barrier to oxidation, including vitamin C, vitamin E, uric acid, bilirubin and others, are responsible for this biological antioxidant potential.^24^, ^25^, ^26^ This study aimed to quantify and compare the inflammatory (IL‐6 and PON‐1) and perioxidative (BAP and derivatives of d‐ROMs) responses resulting from elective ovariectomy performed via the classical open‐air (OPEN) and laparoscopic techniques (LAP) in anestrus dogs. The hypothesis was that LAP would induce less inflammation and OS, supporting the use of minimally invasive surgery for elective ovariectomy in dogs.

## 2 MATERIALS AND METHODS

All procedures were approved by the The Animal Ethics Committee of the University of Naples Federico II (ethical clearance no. 51877‐2023). Informed consent was submitted to the owner of each dog, accepted, and signed.

This prospective blinded, randomized, clinical trial aimed to assess inflammatory and OS responses in bitches subjected to OPEN versus LAP. It was conducted according to consolidated standards of reporting trials (CONSORT) guidelines.^27^

A total of 30 dogs referred between January and December 2024 to Veterinary Teaching Hospital (VTH) of the University of Naples Federico II for elective ovariectomy were prospectively evaluated for study eligibility. The inclusion criteria were as follows: (i) age ≥ 12 months; (ii) bodyweight between 10 and 20 kg; (iii) ASA (American Society of Anesthesiologists) −I physical status classification; and (iv) confirmed to be in anestrus, as determined by colpocytology and serum progesterone concentrations. Dogs were excluded if they presented with concurrent disease; behavioral issues that made handling difficult; preoperative anesthetic complications; experienced major anesthetic or postoperative complications; or they deviated from the standardized anesthetic or surgical protocol.

After recruitment, 26 dogs were randomly assigned to one of two groups (13 from each group): the OPEN group, which underwent conventional laparotomic ovariectomy, or the LAP group, which underwent ovariectomy via a two‐cannula laparoscopic technique. Random allocation of animals to either Group LAP or Group OPEN was performed using a random number generator (Excel, Microsoft Corporation). Allocation concealment was maintained until the day of surgery. After surgery, all dogs were hospitalized for 24 h, and a postoperative follow‐up visit was scheduled for 7 days later. To evaluate differences in inflammatory and oxidative status between the two techniques, the serum concentrations of IL‐6, PON‐1, BAP and d‐ROMs were measured at the following time points: 1 h before the beginning of surgery (Tpre), 2 h after surgery (T2), 24 h post‐surgery (T24), and 7 days post‐surgery (T7).

Surgeons assigned a number to each serum sample and were the only ones who knew the correspondence between the number, the dog, and the procedure performed. Therefore, the investigators performing the biomarker analyses were blinded to group allocation. The surgeons were not blinded due to the nature of the procedures.

### 2.1 Colpocytological examination and serum progesterone (P4) measurements

Anestrus was identified by the presence of more than 80% parabasal cells, a few intermediate cells, an absence of anucleate superficial cells, and minimal to no neutrophils on the vaginal cytology smear, along with serum progesterone concentrations of ≤1 ng/mL (≤4 nmol/L).

Cytology was performed on vaginal cells collected by inserting a cotton‐tipped swab into the dorsal commissure of the vulva, gently advancing in a craniodorsal direction until it passed over the ischial arch, rotating it in both directions and then withdrawing it. The cotton swab was rolled across a glass microscope slide, and the smears were fixed with ethanol and subsequently stained via the Diff‐quick protocol (Bio‐Optica, Milan, Italy). Smear evaluation was independently performed by two experts, each with over 10 years of documented experience in the field. A minimum of 10 fields were examined under a light microscope (Leica DMLB; Wetzlar, Germany) at magnifications ranging from 100× to 400×. Cell classification was performed according to previously established criteria.^28^, ^29^

Progesterone concentrations were measured via a catalyst assay (Catalyst R Progesterone, IDEXX Laboratories Inc., Westbrook, Maine) run on a Catalyst Dx Chemistry Analyzer (IDEXX Laboratories, Inc.) following the manufacturer's instructions.

### 2.2 Anesthesia and analgesia protocols

Dogs were premedicated with a combination of dexmedetomidine (3 μg/kg; Sedadex, Dechra, Torino, Italy) and methadone (0.2 mg/kg; Semfortan, Dechra) intramuscularly (IM). The cephalic vein was aseptically catheterized, and 5 mL/kg/h Lactated Ringer's solution was infused. Dogs were preoxygenated via a facemask for 5 min before the induction of anesthesia was achieved, with intravenous (IV) propofol (Propomitor, Orion Pharma, Milano, Italy) administered to effect. After intubation, with appropriately sized cuffed endotracheal tubes, the animals were connected to a rebreathing circuit coupled to an anesthetic machine (Wato EX‐35Vet, Mindray Medical Italy S.R.L., Trezzano sul Naviglio, Milano, Italy), and anesthesia was maintained with isoflurane vaporized in a mixture of oxygen and medical air (60% oxygen). The respiratory rate, heart rate (obtained from a continuous lead II electrocardiography recording), hemoglobin oxygen saturation, noninvasive arterial blood pressures, end‐tidal carbon dioxide, end‐tidal isoflurane concentrations, and temperature were continuously monitored via a multiparametric monitor (iPM 12Vet, Mindray Medical Italy S.R.L).

### 2.3 Surgical procedures

Both the laparoscopic and open procedures were performed by the same team of experienced surgeons, whose expertise in the field is supported by a substantial documented surgical caseload.

#### 2.3.1 Laparoscopic ovariectomy

LAP surgeries were performed via a two‐portal technique with midline access as previously described.^27^ The dogs were placed in dorsal recumbency and the reverse Trendelenburg position. The area from the xiphoid to the pubis was aseptically prepared. The first trocar (5 mm) was placed via the modified Hasson technique^30^: briefly, a 6 mm incision was made 1 to 2 cm cranial to the umbilicus through the skin and subcutaneous tissue down to the linea alba. The linea alba was cut precisely for inserting the trocar into the abdomen under direct vision. The abdomen was insufflated with CO_2_ to create pneumoperitoneum by connecting the trocar to high‐flow insufflators (Endoflator 264 305 20 Karl Storz, Tuttlingen, Germany) using carbon dioxide until a pressure between 8 and 12 mmHg was achieved. A 5 mm diameter 30° angle of vision rigid laparoscope (HOPKINS, Karl Storz) connected to a light source was inserted into the abdomen, and a 360° scan was performed to check for any existing abnormalities. A 10 mm skin incision was made midway between the umbilicus and pubis, and the second 10 mm portal was inserted under direct visualization to prevent injury to the abdominal organs. The second trocar (10 mm) was introduced cranially to the bladder. After general abdominal exploration, the dog was placed into left lateral recumbency to remove the right ovary and subsequently into right lateral recumbency to remove the left ovary. To perform the gonadectomy, the proper ligament of the ovary was grasped with 5 mm Babcock forceps (Click Line grasping forceps, 5 mm, 36 cm, Karl Storz), elevated, and temporarily secured to the body wall by passing percutaneously a 5 cm, 3/8 circle curved cutting needle. A 5 mm bipolar vessel sealing device (Enseal Trio Device, Johnson & Johnson Medical) was used to coagulate and cut the ovarian pedicles, the suspensory ligament, and the uterine horn. Once the absence of bleeding was verified, the grasping forceps holding the resected ovary was brought directly towards the camera while the camera was simultaneously pulled backwards until the forceps entered inside the caudal port cannula and were observed from the outside. The cannula was subsequently removed, and the ovary was removed from the abdomen. After both ovaries were removed, the abdomen was scanned to ensure hemostasis or any other complications. Each abdominal portal was closed in 3 layers. The abdominal wall was closed using polyglactin 910 (Vicryl 2–0, Ethicon, Norderstedt, Germany). The subcutaneous tissue and skin were closed using poliglecaprone 25 (Monocryl Plus 3–0, Ethicon, Norderstedt, Germany). All three layers were sutured in a simple interrupted pattern.

#### 2.3.2 Open ovariectomy

The OPEN procedure was performed as previously described^31^ with the dog in dorsal recumbency. A midline ventral incision, approximately 2–3 cm in length, is made just caudal to the umbilicus and extending caudally. The linea alba was incised to access the abdominal cavity. The ovaries were identified by following either the left or right uterine horn proximally and exteriorized using atraumatic forceps. The suspensory ligament was disrupted to facilitate exposure. The ovarian pedicle was hemostatized using a bipolar 5 mm vessel sealing device (Enseal Trio Device, Johnson & Johnson Medical, Roma, Italy), which was then checked for hemorrhage. The uterine horn was released into the abdomen. The procedure was repeated for the second ovary. Finally, the abdominal incision was closed in a routine three‐layer manner. The linea alba was closed using polyglactin 910 (Vicryl 2–0, Ethicon, Norderstedt, Germany). The subcutaneous tissue and skin were sutured using poliglecaprone 25 (Monocryl Plus 3–0, Ethicon, Norderstedt, Germany). All three layers were closed in a simple interrupted pattern.

### 2.4 Surgical time and incision length

Surgical time was calculated as the interval from the initial skin incision to the placement of the final closing suture. The incision length was measured at the end of surgery via a Jameson caliper (Karl Storz). The total length of both LAP incisions was compared to the length of the OPEN incision.

### 2.5 Blood samples and analysis

Venous blood samples (4 mL) were collected via jugular venipuncture into 3‐mL plastic serum tubes (Sarstedt AG & Co., Nümbrecht, Germany). Within 30 min of collection, the serum was separated by centrifugation at 2000 × g for 10 min and stored at −80°C for later analysis.

#### 2.5.1 Evaluation of inflammatory markers (IL‐6 and PON‐1)

The concentration of IL‐6 was measured via a commercially available kit according to the manufacturer's instructions (Quantikine Canine IL‐6 Immunoassay CA 6000; R&D Systems, Minneapolis).

Serum paraoxonase‐1 (PON‐1) activity was measured via the enzymatic method^32^ and quantified spectrophotometrically with a SAT450 analyzer (KPM Analytics, Westborough, Massachusetts). PON‐1 activity is expressed in U/mL.

#### 2.5.2 Evaluation of oxidative stress (BAP and d‐ROMs)

OS and antioxidant response capacity were assessed via a validated method that quantified BAP and d‐ROMs, employing commercial Diacron kits (Grosseto, Italy), according to the manufacturer's instructions, and analyzed with a SAT450 analyzer (KPM Analytics). These methodologies, previously described in dogs by Pasquini et al. and Rubio et al,^23^, ^33^, ^34^ express d‐ROMs in UCARR and BAP in μmol/L with reported normal reference ranges of 56.4–91.4 UCARR for d‐ROMs and 1440–3260 μmol/L for BAP.

### 2.6 Statistical analysis

The Shapiro–Wilk test was used to assess the normality of the data. Power analysis for detecting differences between two independent means was performed using G*Power software (version 3.1.9.7; Düsseldorf, Germany). The tests were two‐tailed, and the effect size (Cohen's *d*) was calculated based on the means and standard deviations of the two groups. A post hoc calculation of statistical power was subsequently performed, incorporating the standard deviations of each group for each marker at each time point.^32^ Owing to the small sample size and nonnormal data distribution, nonparametric tests were applied. All the results are presented as median values and interquartile ranges (IQRs). Comparisons of the continuous variables between the LAP and OPEN groups at each time point were conducted via the Mann–Whitney U test. Within‐group comparisons across time points were performed via the Wilcoxon signed‐rank test. All the statistical analyses were performed via SPSS (version 29.0, IBM Corp., Armonk, New York), with the significance threshold set at *p* ≤ .05.

## 3 RESULTS

A total of 26 dogs met all inclusion criteria and were in anestrus based on their vaginal cytological profile and serum progesterone levels. With 13 dogs per group, the study achieved >80% power to detect differences between the OPEN and LAP groups for IL‐6 and d‐ROMs across all time points. In contrast, power for PON‐1 and BAP ranged from 15% to 99%, exceeding 80% only where BAP differed between groups and for PON‐1 at T24.

Dogs included in the study were aged between 12 and 18 months (median: 15 months) and weighed between 10 and 20 kg (median: 18 kg). There were no differences between the groups with respect to age and weight (*p* = .085 and *p* = .701, respectively).

### 3.1 Surgical time and incision length

The duration of the procedure was longer for the LAP group (median: 35 min; range: 30–45 min) than for the OPEN group (25 min; 22–43 min; *p* = .008). The length of the incision was greater in the OPEN group (LAP 1.5 cm; range: 1–2.5 cm; OPEN 2.5 cm; range: 2–3.5 cm; *p* < .001). No dogs experienced surgical and/or anesthesiologic complications during or after the surgery.

### 3.2 Inflammatory markers

#### 3.2.1 IL‐6 and PON‐1

**FIGURE 1:**
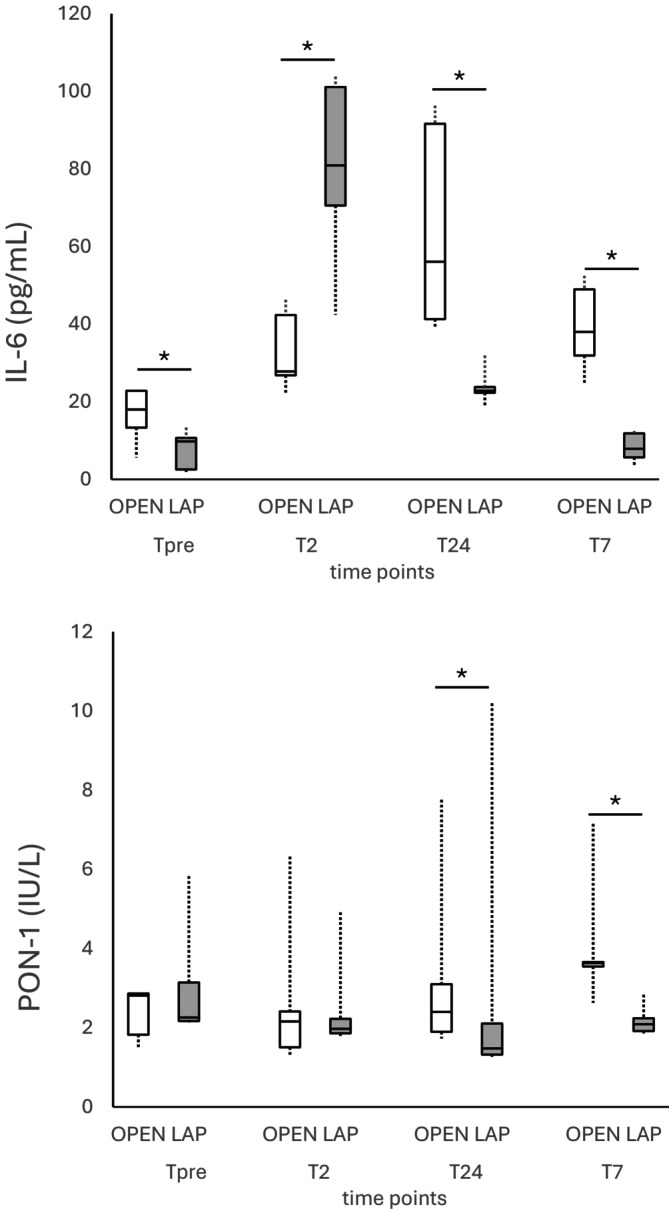
Intergroup differences (*p < .05) between the OPEN (white) and LAP (gray) groups at each time point (Tpre = presurgery; T2 = 2 h post‐surgery; T24 = 24 h post‐surgery; T7 = 7 days post‐surgery) of IL‐6 and PON‐1 concentrations (*n* = 13). For each box, the central line represents the median, the edges represent the interquartiles (IQRs) (25th and 75th percentiles), and the whiskers represent the extreme points.

The levels of the inflammatory markers IL‐6 and PON‐1 are presented in Figure 1.

The concentrations of IL‐6 were greater (*p* < .05) in the OPEN group than in the LAP group at Tpre, T24, and T7 after surgery; moreover, the IL‐6 concentration in the LAP group was greater (*p* < .05) than that in the OPEN group 2 h after surgery.

In the OPEN group, the IL‐6 concentration increased (*p* < .05) at T2, with a further increase (*p* < .05) at T24, followed by a decrease (*p* < .05) at T7. In contrast, the LAP group presented an increase in IL‐6 (*p* < .05) concentrations at T2, but a decrease was already evident (*p* < .05) by T24, with concentrations returning to preoperative levels by T7.

The PON‐1 concentrations differed between the two techniques at T24 (*p* < .05) and T7 (*p* < .001), with lower values observed in the LAP group than in the OPEN group.

In the OPEN group, PON‐1 levels decreased (*p* < .05) at T2, followed by an increase (*p* < .05) at T24 and T7. Moreover, the LAP group presented a decrease (*p* < .05) in PON‐1 at T2, which remained relatively stable at T24 and T7.

### 3.3 Oxidative stress markers (BAP and d‐ROMs)

**FIGURE 2:**
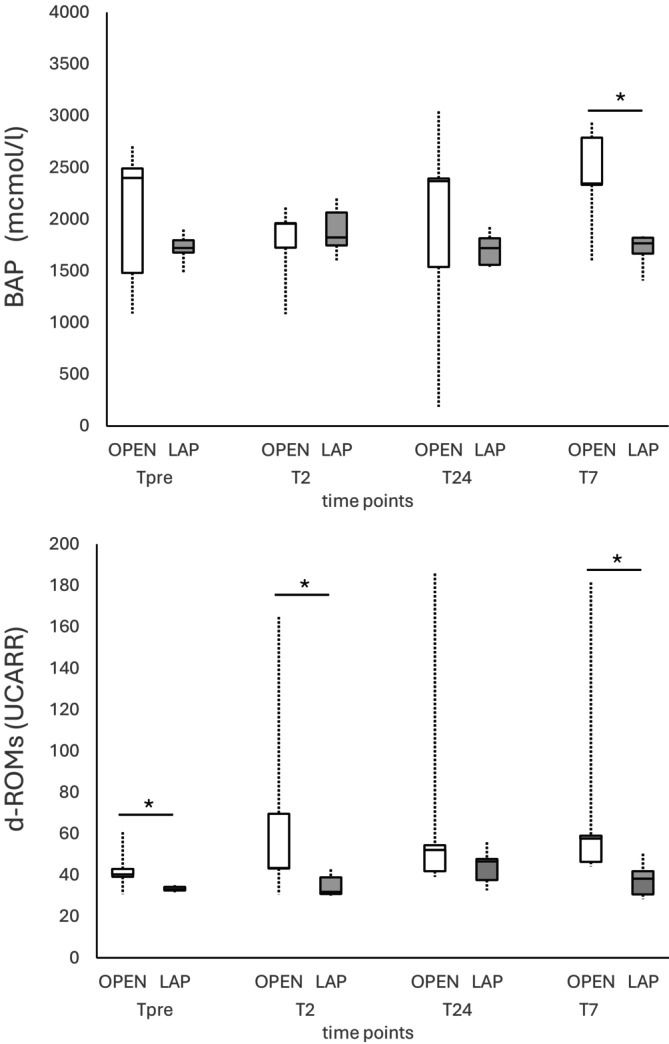
Intergroup differences (*p < .05) between the OPEN (white) and LAP (gray) groups at each time point (Tpre = presurgery; T2 = 2 h post‐surgery; T24 = 24 h post‐surgery; T7 = 7 days post‐surgery) of biological antioxidant potential (BAP) and reactive oxygen metabolites (d‐ROMs) (*n* = 13). For each box, the central line represents the median, the edges represent the interquartiles (IQRs) (25th and 75th percentiles), and the whiskers represent the extreme points.

No differences in BAP concentrations were detected between the OPEN and LAP groups until T7, when the OPEN group presented higher (*p* < .05) concentrations than did the LAP group. Within the OPEN group, the BAP concentrations at T7 were greater (*p* < .05) than those at Tpre and T2. In the LAP group, a difference (*p* < .05) in BAP concentration was observed only between T2 and T7 (Figure 2).

Between‐group differences in d‐ROM concentrations were observed at Tpre, T2, and T7 (*p* < .05), with higher values for the OPEN group. In the OPEN group, the d‐ROM concentration increased at T7. In contrast, the LAP group showed an increase at T24, followed by a decrease (*p* < .05) at T7, with concentrations returning to preoperative levels (Figure 2).

## 4 DISCUSSION

This the first study to simultaneously compare the inflammatory response and OS in female dogs in anestrus following LAP and OPEN ovariectomy.

Selecting a narrow range of age and weight allowed us to obtain a homogeneous group, thereby reducing bias due to the small sample size. The age criterion was consistent with our hospital policy, which, in accordance with the literature, generally considers the age range of 12–24 months as the most appropriate for elective ovariectomy.^35^, ^36^ Similarly, only ASA I dogs were selected in order to avoid pharmacological and metabolic interferences with the inflammatory response to surgery. Furthermore, elective ovariectomies are more commonly performed in young and healthy dogs, according to the literature.^1^ The results demonstrated no differences between groups in age or bodyweight, confirming the homogeneity of the study population.

The inclusion of only dogs in anestrus eliminates the variability due to the endocrine reproductive status and standardizes the sample based on reproductive cycle phase, reducing discrepancies previously reported in the literature. Previous similar studies did not include the estrous cycle phase as a selection criterion, representing a critical methodological omission. Sex hormones profoundly affect both inflammation and oxidative homeostasis in both humans and dogs.^37^ Estrogen and progesterone are recognized as anti‐inflammatory substances that can modulate the polarization of immune cells and suppress proinflammatory cytokines, thereby supporting the resolution of inflammation and tissue repair.^38^ Moreover, the hypothalamic–pituitary–adrenal and hypothalamic–pituitary–gonadal axes regulate the canonical postsurgical cytokine cascade.^37^ Recently, IL‐6 has been recognized as a useful marker for assessing inflammation in the postoperative period, even in healthy bitches undergoing sterilization.^39^ However, to date, no study has directly investigated the influence of circulating sex hormones on the comparison of postoperative inflammatory dynamics in bitches undergoing LAP and OPEN. Animal models of ischemia–reperfusion injury suggest that removal of endogenous sex hormones intensifies local inflammation, presumably due to loss of hormonal protection.^33^ During the canine estrus cycle, the balance between inflammation and OS undergoes physiological changes, modifying the concentrations of IL‐6, PON‐1, and OS biomarkers.^35^, ^36^, ^39^

Previous studies have indicated that IL‐6, PON‐1, BAP, and d‐ROMs may be influenced by storage time and temperature to varying degrees, emphasizing the importance of standardized preanalytical procedures.^13^, ^33^ In the present study, particular care was taken to minimize such variability and serum samples were stored at −80°C until analysis, in accordance with established recommendations for optimal biomarker preservation.^13^, ^33^ Therefore, although the influence of sample handling remains a recognized methodological concern in biomarker studies, it is unlikely to have affected the present results.

Open ovariectomy or ovariohysterectomy in bitches induced a transient acute inflammatory response, evidenced by an increase in IL‐6. In traditional surgery, IL‐6 already increases within 6–30 h, together with C‐reactive protein and other inflammatory markers.^39^, ^40^ Additionally, PON‐1 remains a useful biomarker for evaluating post‐surgical inflammation in dogs.^41^, ^42^ IL‐6 has been employed as a comparative inflammatory marker in canine studies evaluating laparoscopic versus open procedures.^43^ Indeed, a prior study demonstrated a smaller increase in IL‐6 at 24 h with laparoscopic ovariectomy.^43^

In this study, IL‐6 in the LAP group exhibited an early but transient and self‐limiting increase. Conversely, in the OPEN surgery group, IL‐6 levels peaked later and remained elevated for an extended period, persisting at higher levels up to 7 days after surgery. A rapid increase in proinflammatory cytokines soon after laparoscopy has already been observed in other studies and can be attributed to intra‐abdominal pressure related to CO_2_ insufflation which can cause visceral pain and peritonitis.^44^ However, this increase in IL‐6 in LAP surgeries is self‐limiting, decreasing within 24 h and returning to physiological levels within 7 days after surgery. Furthermore, in the present study, the LAP procedures took longer than the OPEN ones; however, the latter involved longer incisions. Our results are in line with previous results and support the hypothesis that minimally invasive procedures offer advantages over open surgery by attenuating the overall inflammatory response.^43^, ^44^

This rapid effect of LAP surgery on the inflammatory response, and consequently on oxidative stress, is further supported by the trends observed in PON‐1 concentrations. Although PON‐1 is mainly recognized for its antioxidant function, in our methods it was classified among inflammatory markers because its serum activity is inversely affected by the inflammatory state. PON‐1 is synthesized in the liver and bound to HDL particles, which play a crucial role in maintaining its stability and activity in circulation.^45^ When the organism experiences only a mild inflammatory insult, hepatic synthesis of PON‐1 is not affected, as the levels of proinflammatory cytokines such as IL‐6 and TNF‐α are insufficient to markedly decrease its production or alter HDL composition. Consequently, PON‐1 levels remain relatively high and continue to exert their functions. In some cases, a slight increase in PON‐1 activity may even occur as a compensatory response.^46^, ^47^ The role of PON‐1 as a biomarker in surgical settings in dogs remains poorly defined. The scientific literature shows that in dogs the activity of PON‐1 decreases in response to different systemic inflammatory conditions, such as sepsis, trauma and acute pancreatitis.^42^, ^43^ These decreases have been associated with high levels of traditional inflammatory markers, reinforcing the inverse relationship between inflammation, oxidative stress and PON‐1 activity. On the contrary, Rossi et al. did not find a decrease in PON‐1 under experimental inflammatory conditions, despite observing increases in other biological markers such as C‐reactive protein and α_2_‐globulin.^13^ However, to date, no published study has directly compared PON‐1 responses in dogs between LAP and OPEN. In this study, PON‐1 concentrations decreased postoperatively in both groups but remained lower only in the LAP group at T24 and T7.

PON‐1, indeed, is a liver‐derived, HDL‐associated enzyme recognized as a negative acute‐phase protein in both human and veterinary medicine. Nevertheless, its diagnostic reliability as an inflammatory biomarker in dogs remains controversial. In the present study, both experimental groups exhibited PON‐1 activities below the reported reference range,^13^ with a greater reduction in the LAP group that persist until 7 days. The PON‐1 downregulation during all the experimental times is probably due to the postoperative peak of IL‐6; indeed, the PON‐1 activity typically returns to normal within a 3–7 days.^48^ These findings are consistent with previous reports and support the notion that PON‐1 may act as a complementary, rather than primary, indicator of systemic inflammation in dog.

Video‐assisted surgery, while proven to induce less inflammatory response than open surgery, can induce more oxidative stress due to pneumoperitoneum, which can cause ischemia–reperfusion and production of ROS.^49^, ^50^ However, some studies report conflicting results, with similar levels of OS between the two techniques,^8^ highlighting the need for further research. Our study is the first to employ both BAP and d‐ROMs for assessing oxidative stress in this surgical context. This study shows that the response to OS varies between OPEN and LAP surgery. Levels of d‐ROMs were higher in the OPEN group throughout the postoperative period, peaking at 7 days, suggesting a more prolonged oxidative response. In the LAP group, however, only a transient peak was observed at 24 h, followed by a rapid return to baseline values.

BAP levels remained similar between groups until day seven, when there was an increase in the OPEN group, indicative of a compensatory antioxidant response to a higher oxidative load. This suggests that the LAP approach causes a more restrained and temporary oxidative response.^7^, ^8^ Finally, differences between studies may depend on factors such as the duration of the intervention and the degree of tissue trauma, directly affecting the inflammatory and oxidative response.

The main limitation of the present study was the small sample size, as demonstrated by the post hoc power analysis for PON‐1 and BAP values, in which the statistical power was below 80% at several time points. Although the statistical power for IL‐6 and d‐ROMs was high across all time points, and the power for BAP was adequate at the time points where differences were observed, a larger sample size could have provided greater robustness and relevance to the differences detected.

In conclusion, our findings demonstrated a reduced inflammatory response and a less pronounced oxidative imbalance in bitches treated with LAP than in those treated with the OPEN approach. Our study employed for the first time in this surgical context validated markers of inflammation and OS. These results help to objectify and clarify previously discordant data in the literature by homogenizing the study population, including only animals in anestrus, and eliminating the potential confounding variable of circulating gonadal hormones. Taken together with existing literature, these findings provide additional support for minimally invasive surgery as a low‐trauma technique for elective ovariectomy in dogs.

## AUTHOR CONTRIBUTIONS

Calabria A, DVM, PhD: Contributed to the study's design, participated in surgical procedures, compiled all data, interpreted data, and drafted and revised the manuscript. Del Prete C, DVM, PhD: Contributed to the study's design, performed surgical procedures, and drafted and revised the manuscript. Pasolini MP, DVM, PhD: Analyzed and interpreted data for statistical significance, revised the manuscript. Palumbo V, DVM, PhD: Contributed to the design of the study, performed anesthesia, evaluated pain scales, and interpreted data. Nappo MA, DVM: Performed surgical procedures and drafted and revised the manuscript. Ammirati S, DVM: Analyzed data for statistical significance and drafted and revised the manuscript. Davide Ciccarelli, DVM, PhD: Performed laboratory analysis and analyzed data for statistical significance. Micieli F, DVM, PhD: Performed anesthesia, evaluated pain scales, and interpreted data. Kosior MA, DVM, PhD: Contributed to the study's design and analyzed data for statistical significance. Carbone C, DVM: Contributed to the design of the study, interpreted data, and provided scientific, in‐line editing of the manuscript. Cocchia N, DVM, PhD: Design the study, was responsible for the surgical management of the case, data collection, and intraoperative photographs, interpreted data, drafted and revised the manuscript, and provided scientific, in‐line editing of the manuscript. All co‐authors provided a critical review of the manuscript, submission of revisions, and final approval of proofs.

## CONFLICT OF INTEREST STATEMENT

The authors declare that the research was conducted in the absence of any financial relationship that could be construed as a potential conflict of interest.

## Data Availability

The data that support the findings of this study are available from the corresponding author upon reasonable request.
